# Transcriptome profiling of pediatric extracranial solid tumors and lymphomas enables rapid low-cost diagnostic classification

**DOI:** 10.1038/s41598-024-70541-0

**Published:** 2024-08-21

**Authors:** Kofi B. Opoku, Teresa Santiago, Priya Kumar, Sophia M. Roush, Yuri Fedoriw, Tamiwe Tomoka, Vasiliki Leventaki, Larissa V. Furtado, Nickhill Bhakta, Thomas B. Alexander, Jeremy R. Wang

**Affiliations:** 1 Department of Genetics, University of North Carolina at Chapel Hill, Chapel Hill, USA; 2 Department of Pathology, St. Jude Children’s Research Hospital, Memphis, USA; 3 Department of Pathology and Laboratory Medicine, School of Medicine, University of North Carolina at Chapel Hill, Chapel Hill, USA; 4 Department of Pathology and Laboratory Medicine, Lineberger Comprehensive Cancer Center, University of North Carolina at Chapel Hill, Chapel Hill, USA; 5 University of Malawi College of Medicine, Blantyre, Malawi; 6 University of North Carolina at Chapel Hill, Chapel Hill, USA; 7 UNC Project Malawi, Lilongwe, Malawi; 8 Department of Hematopathology, University of Texas MD Anderson Cancer Center, Houston, USA; 9 Department of Global Pediatric Medicine, St. Jude Children’s Research Hospital, Memphis, USA; 10 Department of Pediatrics, Lineberger Comprehensive Cancer Center, University of North Carolina at Chapel Hill, Chapel Hill, USA

**Keywords:** Cancer genomics, Machine learning, Paediatric cancer, Translational research

## Abstract

Approximately 80% of pediatric tumors occur in low- and middle-income countries (LMIC), where diagnostic tools essential for treatment decisions are often unavailable or incomplete. Development of cost-effective molecular diagnostics will help bridge the cancer diagnostic gap and ultimately improve pediatric cancer outcomes in LMIC settings. We investigated the feasibility of using nanopore whole transcriptome sequencing on formalin-fixed paraffin embedded (FFPE)-derived RNA and a composite machine learning model for pediatric solid tumor diagnosis. Transcriptome cDNA sequencing was performed on a heterogenous set of 221 FFPE and 32 fresh frozen pediatric solid tumor and lymphoma specimens on Oxford Nanopore Technologies’ sequencing platforms. A composite machine learning model was then used to classify transcriptional profiles into clinically actionable tumor types and subtypes. In total, 95.6% and 89.7% of pediatric solid tumors and lymphoma specimens were correctly classified, respectively. 71.5% of pediatric solid tumors had prediction probabilities > 0.8 and were classified with 100% accuracy. Similarly, for lymphomas, 72.4% of samples that had prediction probabilities > 0.6 were classified with 97.6% accuracy. Additionally, *FOXO1* fusion status was predicted accurately for 97.4% of rhabdomyosarcomas and *MYCN* amplification was predicted with 88% accuracy in neuroblastoma. Whole transcriptome sequencing from FFPE-derived pediatric solid tumor and lymphoma samples has the potential to provide clinical classification of both tissue lineage and core genomic classification. Further expansion, refinement, and validation of this approach is necessary to explore whether this technology could be part of the solution of addressing the diagnostic limitations in LMIC.

## Introduction

Low and low-middle Sociodemographic Index (SDI) countries have seen an 81.2% increase in reported prevalence of cancer over the last 30 years despite global reduction in cancer prevalence^1,2^. Noteworthy is the disproportionate increase of reported cancer prevalence by about 73% in Sub-Saharan Africa, where diagnostic tools and treatment options are limited by cost and availability^1–6^. While accounting for only 1% of the global burden of cancer, cancers in children aged 0–19 years account for 11.5 million disability adjusted life years (DALYs) globally, with about 90% of these cases occurring in low- and middle-income country (LMIC) settings^1–3, 7^. Pediatric tumors require highly accurate and specific diagnoses to inform treatment decisions^8^.

Traditionally, accurate diagnosis of pediatric solid tumors involves the use of multiple resource-intensive tests, including different imaging modalities, immunohistochemistry, chromosome-based tests such as karyotype and fluorescence in-situ hybridization (FISH), and molecular testing such as PCR and targeted gene sequencing^8^. Many LMIC settings lack access to this full array of testing, thus reducing diagnostic capability and compromising the selection of the most effective treatment^5,9^.

Established short-read sequencing techniques have shown that pediatric non-CNS solid tumors and lymphomas have unique gene expression profiles (GEP)^10–14^. However, capital and operational costs associated with traditional high-throughput sequencing-based diagnostics prohibit their utility in cancer diagnostics in resource limited settings^10,13–15^.

Nanopore-based sequencing platforms are available at dramatically lower capital cost compared to traditional, more widely used short-read sequencing technologies^14^, enabling direct sequencing of DNA, RNA, and PCR-amplified cDNA on a low-cost portable platform. While extremely long reads are achievable by nanopore sequencing, the platform is equally capable of sequencing nucleic acid fragments as short as 20 nucleotides efficiently^16^.

We previously demonstrated a nanopore transcriptome sequencing approach to characterize lineage and genomic subtypes of pediatric acute leukemia^17^. We have now modified and extended our previous approach to include pediatric solid tumors and lymphomas. Critically, we demonstrated the effectiveness of this approach on RNA derived from formalin-fixed paraffin-embedded (FFPE) samples, since most solid tumors worldwide are stored only as FFPE specimens for clinical diagnosis and research. This widely available preservation method adequately preserves cell architecture and proteins for later examination without cryopreservation, making it ideal for preserving solid tumor samples. However, this method extensively crosslinks nucleic acids causing fragmentation during the extraction process which leads to very short read lengths, typically less than 400 nt. Although the alignment rate of these shorter reads is lower than that of fresh-frozen samples, we demonstrate that they are suitable for robust gene expression profiling and classification of diverse tumor types.

In this study, we investigate the utility of nanopore sequencing and machine learning-based models for accurate classification and subtyping of pediatric extra-cranial solid tumors and lymphomas from FFPE specimens post morphological diagnosis.

## Materials and methods

### Samples

A total of 221 FFPE and 32 fresh-frozen solid tumor specimens (Table 1) collected at the University of North Carolina at Chapel Hill (UNC), St. Jude Children’s Research Hospital (SJCRH), and the University of Wisconsin were included in this study after receiving Institutional Review Board approval from their respective institutions. Paired fresh-frozen and FFPE samples were prepared from 15 xenograft samples, including 5 rhabdomyosarcoma, 4 neuroblastoma, and 6 Ewing sarcoma. All other samples were FFPE-preserved primary tumor resections and in all other cases where multiple samples were sequenced, they represent technical replicates. Samples were de-identified and assigned unique alphanumeric codes linked to deidentified metadata, including pathological diagnosis. Throughout, we use the existing clinical pathological diagnosis (or original diagnostic sample, in the case of xenografts), based on flow cytometry, immunohistochemistry, FISH, microarray, or previous next-generation sequencing as the “ground truth” by which we evaluate our nanopore-based transcriptome approach. A full list of specimens and relevant metadata are available in Supplemental Table S1.

**Table 1:** Pediatric extracranial solid tumors and lymphoma samples.

Tumor type	# Patients	# Samples	FFPE; fresh-frozen
Rhabdomyosarcoma	24	38	28; 10
Neuroblastoma	25	33	25; 8
Ewing sarcoma	24	38	24; 14
Burkitt lymphoma	23	23	23; 0
T-lymphoblastic lymphoma (TLL)	3	3	3; 0
Diffuse large B cell lymphoma (DLBCL)	34	34	34; 0
Wilm’s tumor	12	18	18; 0
Anaplastic large cell lymphoma (ALCL)	9	13	13; 0
Desmoplastic small round cell tumors (DSRCT)	5	10	10; 0
B-cell acute lymphoblastic leukemia (B-ALL)	11	12	12; 0
Classical Hodgkin lymphoma (CHL)	17	17	17; 0
Nodular lymphocyte-predominant Hodgkin lymphoma (NLPHL)	14	14	14; 0

### Transcriptome sequencing

RNA was extracted from FFPE and fresh-frozen samples using Zymo Research’s Quick-DNA/RNA FFPE extraction kit and Zymo Quick RNA Magbead kit, respectively, following the manufacturer’s instructions. One 10 µm or two 5 µm slides/scrolls, stained or unstained, were used for RNA extraction from FFPE samples. RNA was quantified using Qubit fluorometer and fragment lengths assessed by 1% TAE gel electrophoresis and imaging using EtBr. Gel imaging confirmed that FFPE-derived RNA is severely degraded, as expected, and was only performed on a subset of samples. A minimum of 100 ng RNA was used to prepare cDNA libraries. Libraries were prepared for sequencing using Oxford Nanopore Technologies’ (ONT’s) PCR cDNA Barcoding Kit (SQK-PCB109) per the manufacturer’s protocol, or pre-barcoded cDNA was prepared using ONT’s Ligation Sequencing Kit (SQK-LSK110, SQK-LSK112, or SQK-LSK114). Briefly, 100 ng total RNA was reverse-transcribed using Maxima H- Reverse Transcriptase with a dT30VN primer to capture poly-adenylated mRNA (excluding rRNA) and a strand-switching primer to enable subsequent PCR amplification of the full-length cDNA fragments. Second-strand synthesis and PCR (20 cycles) were performed using LongAmp Master Mix (2X) with up to 12 unique barcoded primers. Amplified cDNA products were purified using Ampure XP SPRI beads. Multiplexed libraries were pooled and sequenced using either ONT MinION or P2 sequencing platforms for up to 72 h. Reads were base called and demultiplexed using Guppy (version 6.0 and up) in super-accuracy mode.

### Gene expression quantification

Nanopore reads were aligned to ENSEMBL (GRCh38 v109) mRNA + ncRNA^18^ using Minimap2^19^ with “-x map-ont -k12 -w1 -n2 -m20". Alignment parameters were made more sensitive than the default for nanopore reads to account for short read lengths produced from FFPE-derived RNA/cDNA. These settings dramatically increase the alignment rate for short nanopore reads with a moderate increase in alignment time and spurious alignments. Transcripts were quantified by counting reads aligning to each transcript, assigning partial counts for multiply aligning reads, and normalized to transcripts-per-million (TPM) using custom software (https://github.com/jwanglab/minnow).

### Lineage assignment and molecular classification

We implemented a supervised machine learning model using the same architecture we previously described for leukemia classification^17^. Briefly, ENSEMBL transcript counts are collapsed at the gene level and the gene-level expression matrix is filtered to remove genes with zero expression in > 1% of samples. This is consistent with our previous work showing that sparse expression data resulting from low-coverage sequencing tends to over-fit most prediction models if not aggressively filtered. With this expression matrix, we built a composite model consisting of a set of partial least-squares (PLS) regressions representing each pair of classes (ex. rhabdomyosarcoma vs. Ewing sarcoma) and each class versus all others (ex. rhabdomyosarcoma vs. all other pediatric solid tumors). For each of these sets, we train PLS models including $n\in \left[\text{5,11}\right]$ components. All of the resulting component vectors are then used to train a support vector machine to assign final probabilities to all classes. This composite PLS-SVM model was previously shown to significantly outperform traditional linear models and ordination methods in the context of low-coverage nanopore transcriptomics leading to sparse, heterogeneous gene expression profiles^17^.

### Entropy, correlations, and statistical testing

Shannon entropy^20^ was calculated over the unfiltered transcripts-per-million (TPM) using the Shannon–Wiener diversity index performed in Python using scikit-bio (http://scikit-bio.org). Linear regressions between quality control metrics (number of reads, read length, N50, entropy) were performed in Python with SciPy, including Pearson correlation coefficient (*r* value) and p-value using Wald Test with t-distribution.

### Evaluation and validation

We evaluated our machine learning model on these data using leave-one-out cross-validation, excluding matched technical or biological replicates when testing each sample. To assess classification results, we compare to the standard of care diagnosis made at the respective institution (UNC Hospitals, Wisconsin Children’s, or St. Jude Children’s Research Hospital), including a combination of morphology, immunohistochemistry, FISH, and established molecular diagnostic evidence (ex. targeted PCR).

### Ethics declarations

This work was reviewed and approved by the University of North Carolina at Chapel Hill Institutional Review Board to conform to NIH and UNC Office of Human Research Ethics guidelines. Informed consent was obtained from all participants and/or their legal guardians.

## Results

### Whole transcriptome sequencing can be used to classify FFPE specimens into histologically distinct and clinically actionable pediatric solid tumor types.

**Fig. 1:**
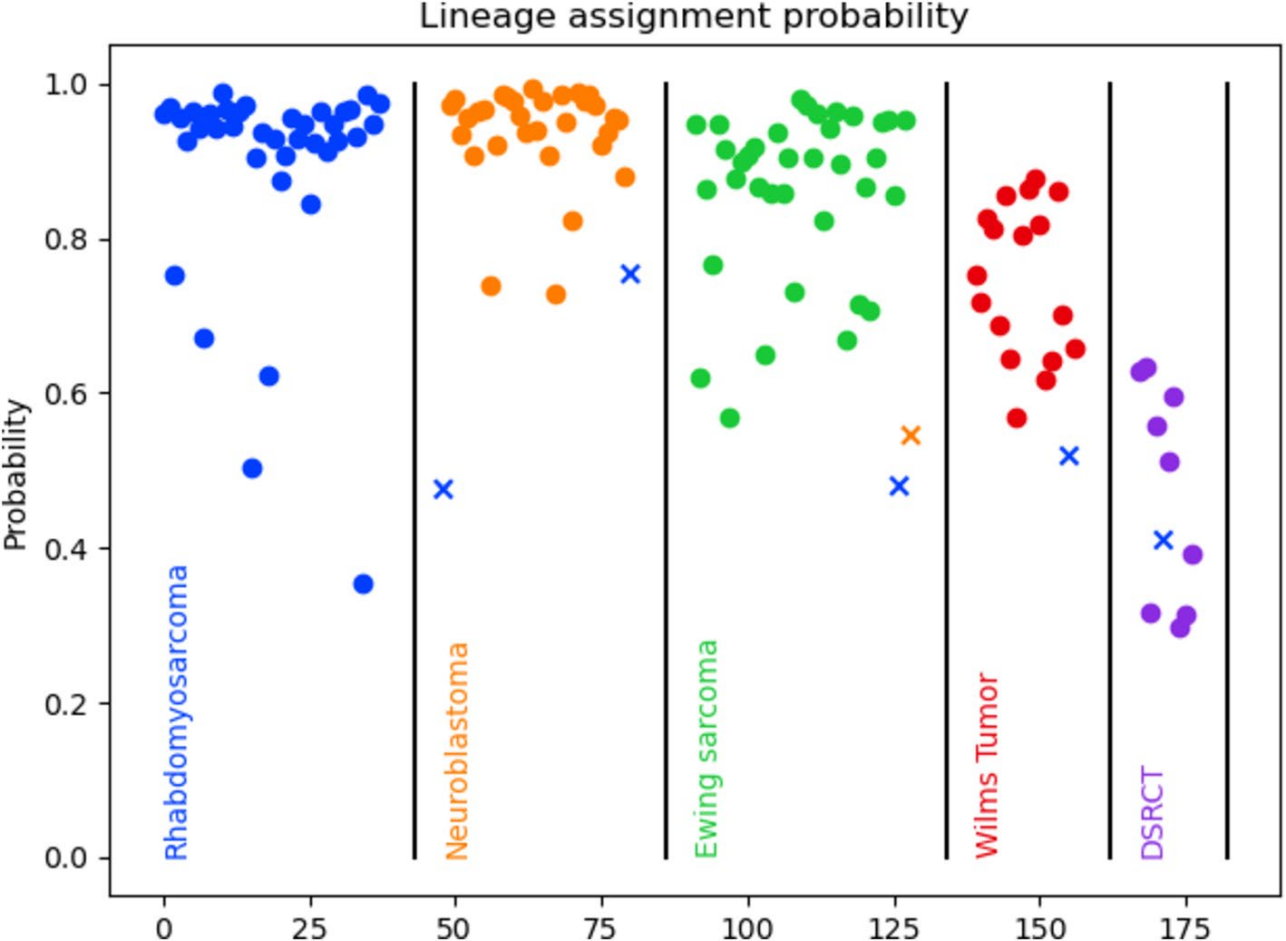
Classification accuracy and prediction probability for pediatric extracranial solid tumors. Each point represents a sequenced sample, with samples arranged by pathological diagnosis (ground truth) along the X axis and prediction probability along the Y axis. Circles are correctly classified and Xs are incorrect and colored according to their predicted tumor type.

We sequenced 137 FFPE specimens of pediatric extracranial solid tumors obtained from 90 patients, including DSRCT, Ewing Sarcoma, Neuroblastoma, Rhabdomyosarcoma, and Wilm’s Tumor (Table 1). To demonstrate the effectiveness of low-coverage (low-cost) nanopore sequencing, we multiplexed up to 12 samples per MinION flow cell and up to 96 per P2 flow cell. Previous work^17^ showed that one sample per Flongle flow cell produces quantitatively and qualitatively similar results. We generated an average of 344,273 reads per sample with an average read length of 251 nucleotides (nt) and read N50 of 286 nt. In total, 131 out of 137 were correctly classified, representing 95.6% accuracy across all solid tumor types tested (Fig. 1, Supplemental Table S2). The classification accuracy by tumor type was 100% (38/38) for rhabdomyosarcoma, 94.7% (36/38) for Ewing sarcoma, 93.9% (31/33) for neuroblastoma, 94.4% (17/18) for Wilms tumor and 90% (9/10) for desmoplastic small round cell tumor (Fig. 1). A corresponding confusion matrix is shown in Fig. S9A. 71.5% of these were called with a prediction probability of > 0.8 with 100% accuracy. Reducing the prediction probability cut off to > 0.6 increases the percentage of calls above that threshold to 88.3% of samples with 99.2% accuracy.

### Whole transcriptome sequencing of FFPE lymphoma specimens can be used to correctly subclassify lymphomas into different lineages to inform clinical direction

We sequenced and analyzed 116 pediatric lymphoma samples as described above. We included 12 FFPE-derived B-ALL/LBL samples because of the clinical challenge of distinguishing mature B- cell lymphoma from lymphoblastic lymphoma/leukemia. Sequenced reads had an average length of 184 nt with an average of 1.12 million reads per specimen.

**Fig. 2:**
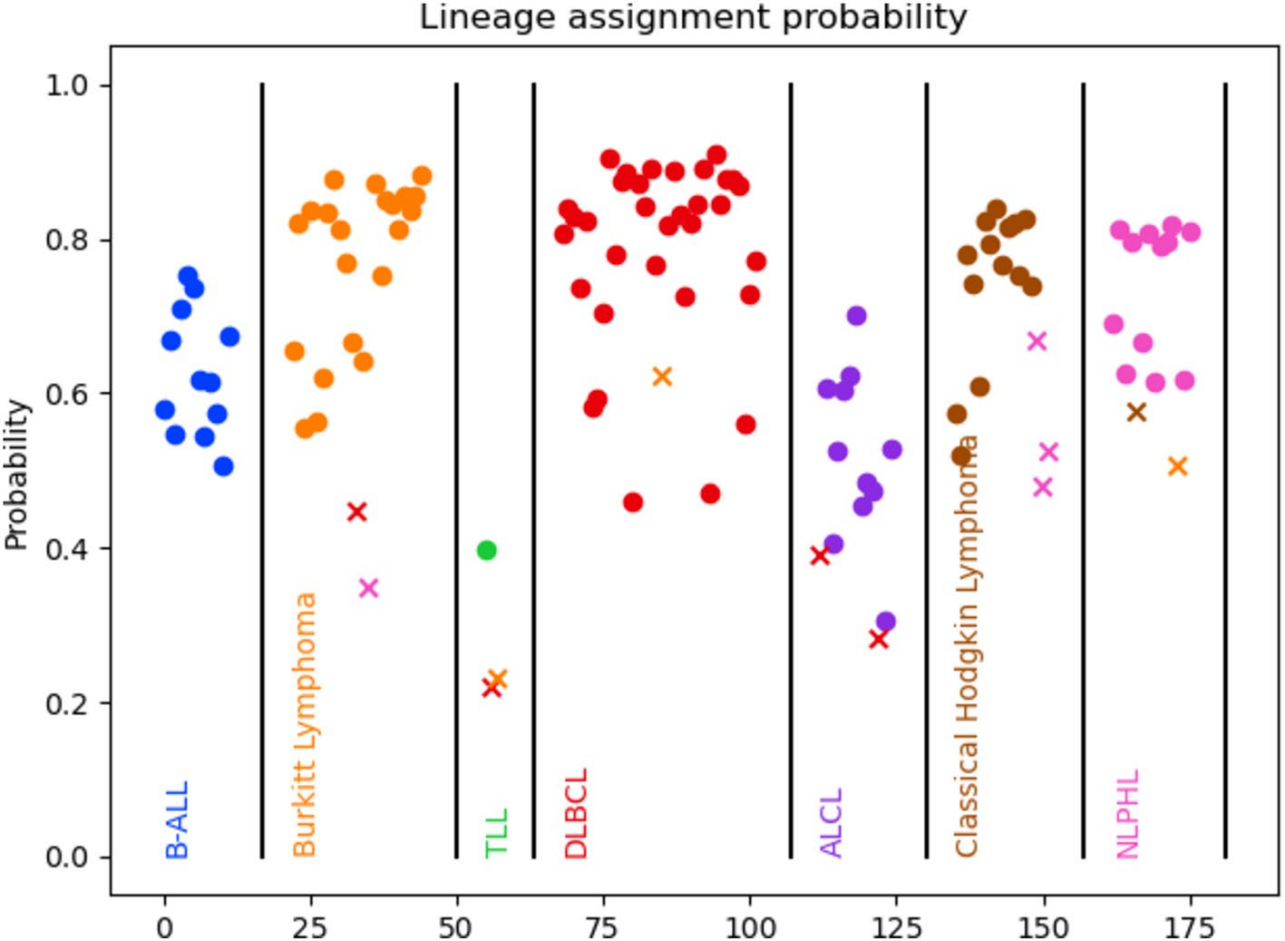
Classification accuracy and prediction probability for pediatric lymphomas. Each point represents a sequenced sample, with samples arranged by pathological diagnosis (ground truth) along the X axis and prediction probability along the Y axis. Circles are correctly classified and Xs are incorrect and colored according to their predicted tumor type.

In total, 104 out of 116 lymphoma specimens were classified correctly, representing an overall accuracy of 89.7% across all tumor types tested (Fig. 2, Supplemental Table S3). By lymphoma type, the accuracy was 84.6% (11/13) for ALCL, 100% (12/12) for B-ALL, 91.3% (21/23) for Burkitt Lymphoma, 82.4% (14/17) for CHL, 97.1% (33/34) for DLBCL, 85.7% (12/14) for NLPHL and 33% (1/3) for T-LBL. A corresponding confusion matrix is shown in Fig. S9B. 37.1% were called with > 0.8 prediction probability with 100% accuracy while 72.4% were called with > 0.6 prediction probability with 97.6% accuracy. Similar to pediatric solid tumors, lymphoma types for which we had fewer specimen numbers had lower prediction probabilities and correspondingly lower accuracy.

### Core genomic and histologic subtypes of rhabdomyosarcoma and neuroblastoma can be determined from whole transcriptome sequencing of FFPE solid tumor specimens

**Fig. 3:**
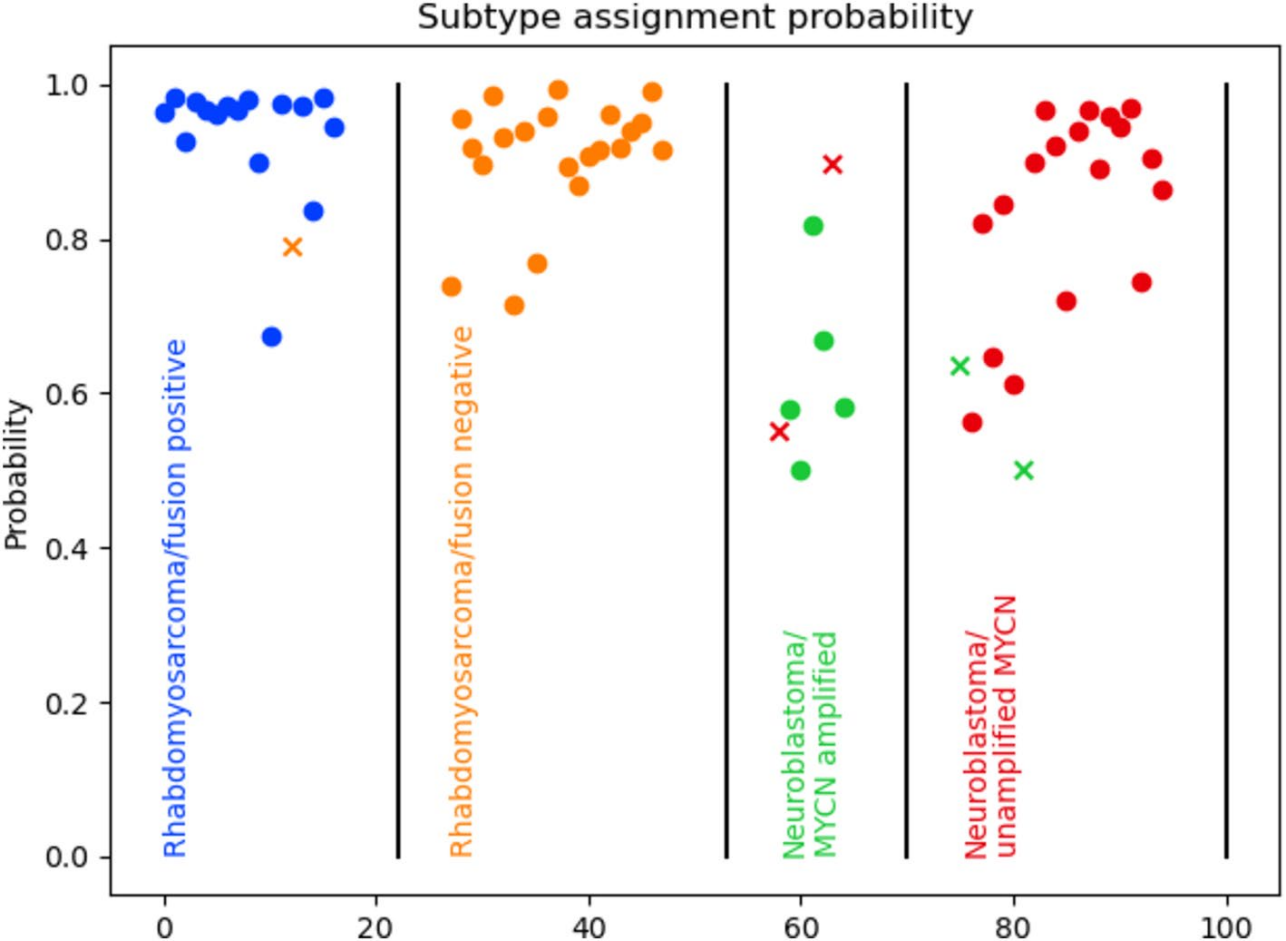
Classification accuracy and prediction probability for rhabdomyosarcoma FOXO1 fusion status (positive or negative) and neuroblastoma MYCN gene amplification status. Each point represents a sequenced sample, with samples arranged by pathological diagnosis (ground truth) along the X axis and prediction probability along the Y axis. Circles are correctly classified and Xs are incorrect and colored according to their predicted subtype.

The fusion status of 97.4% (37/38) of rhabdomyosarcoma specimens were correctly called with prediction probabilities > 0.6 (Fig. 3). We examined the degree of expression of known upregulated/overexpressed genes associated with *FOXO1* fusion-positive alveolar rhabdomyosarcoma (ARMS) versus fusion-negative embryonal rhabdomyosarcoma that have been previously reported^21^. These genes showed a weak relationship with fusion status in our data (Supplemental Fig. S4). We evaluated the differential expression of each of these previously reported genes using a Mann–Whitney U test and found none were significantly differentially expressed in our data after correcting for multiple testing. To identify genes contributing strongly to fusion status predictions in our model, we ordered genes by their coefficient in our PLS regression model (Supplemental Fig. S5), showing clearer differential expression based on fusion status.

The *MYCN* gene amplification status of neuroblastoma specimens was called with 88% accuracy overall, with > 0.5 prediction probability (Fig. 3). Accuracy increases to 90.5% when prediction probability cut-off is raised to > 0.6. Like *FOXO1* fusion status, we saw limited differential expression among genes previously reported to correlate with *MYCN* amplification^22^ (Supplemental Fig. S6). Similar to *FOXO1* fusion status, none were found to be significantly differentially abundant. We report the genes with the highest coefficient in our PLS model (Supplemental Fig. S7).

### Gene expression entropy is correlated with prediction probability and accuracy

To maximize accuracy and increase confidence of diagnostic calls, we evaluated quality control measures such as read N50, number of reads per specimen, Shannon entropy^20^ of gene expression profile, and prediction probability. Both “pass" and “fail" reads (as designated by the Guppy basecaller) were used to assemble the gene expression profile for classification. The only expected difference is a lower alignment rate for lower quality “fail” reads. Read N50, number of reads per specimen, and read length showed no correlation with prediction probability or classification accuracy. However, there was a correlation between Shannon entropy—a measure of transcriptome diversity within each sample—and prediction probability (R^2^ = 0.04; p = 0.0015) (Supplemental Fig. S1). Full sequencing and gene expression quantification metrics are available in Supplemental Table S4.

### Tumor purity has little impact on tumor type prediction

We evaluated the relationship between tumor purity (the proportion of FFPE tissue section that is composed of tumor cells via microscopy) and model performance for a subset of solid tumor samples for which we had reliable tumor purity measurements. Surprisingly, tumor purity was found to be unrelated to the prediction probability or accuracy in our model (Supplemental Fig. S3). The five of these samples that were incorrectly classified had tumor purity values of 40% (1), 60% (1), and 90% (3). We hypothesize that several effects may be working to effectively improve model performance even for lower-purity samples, including similarity in the non-tumor tissue environment among tumors of the same type and non-independence between tumor type and tumor purity. For example, Ewing sarcoma has an average purity of 60.4% in our cohort, but DSRCT and Wilms tumor both average > 90%. This implies that, to the extent that lower tumor purity affects the bulk gene expression profile, these changes themselves represent classifiable signal within our model.

### Chemotherapy treatment status does not impact tumor type prediction

We sought to determine if neoadjuvant chemotherapy for SRBCT alters gene expression in a way that impacts tumor type prediction. Of 76 Wilm’s Tumor, rhabdomyosarcoma, Ewing sarcoma, and neuroblastoma samples with known treatment status, 31 (40.8%) were collected following chemotherapy. Only four (4) of these 76 were misclassified by our model, all of them pre-therapy. Similarly, we saw no significant correlation (p = 0.7) between therapy status and prediction probability (Supplemental Fig. S2).

## Discussion and conclusion

Continued advancement in sequencing technologies have allowed for genomic and transcriptomic characterization of diverse tumor types. These tools have further advanced diagnostic capability, prognostication, and specificity of core genomic types and subtypes of tumors, making it the gold standard for tumor type confirmation and the final step in the pediatric cancer diagnostic testing cascade. Capital and operational costs for the full array of diagnostic tools requisite for accurate diagnosis of pediatric solid tumors including FISH, karyotyping, immunohistochemistry and more established short read sequencing platforms (e.g., Illumina) prohibit their use in resource limited settings, as they are either unavailable, incomplete or just not affordable by patients. The ability to use a low-cost sequencing platform such as ONT’s MinION to sequence FFPE-derived cDNA to accurately classify solid tumors is worth further development due to its potential to eliminate the need for stepwise testing and increase access to diagnostic tools in resource constrained settings, helping to bridge the existing cancer diagnostic gap.

### Classification accuracy and size of training data

Despite the fragmented nature of FFPE derived transcriptomes and the higher depth of sequencing required to improve certainty of mapping and therefore accuracy, we observed an overall accuracy of 95.6%, 89.7% and 97.4% for solid tumors, lymphomas and rhabdomyosarcoma subtype classification respectively, while multiplexing 12 specimens on a single MinION flow cell. Tumor types with a greater number of specimens available for training our model tended to have higher accuracies and prediction probabilities, while those with lower numbers had lower accuracies. The effect is entirely expected and clearly illustrated in Figs. 1 and 2. As an example, T-LBL, for which we had only 3 specimens for testing, showed 33% accuracy with all prediction probabilities being < 0.4. In contrast, 21 out of the 23 Burkitt lymphoma specimens tested had prediction probability > 0.5, with all of these specimens correctly classified.

### Biological and technical replicates

We included in this study several replicates representing different sampling and preservation methods for pediatric solid tumors as well as technical replicates for RNA extraction, library preparation, and sequencing. We processed matched fresh frozen and FFPE samples from xenografts for rhabdomyosarcoma, Ewing sarcoma, and neuroblastoma. Among this subset, only one was incorrectly classified—a fresh frozen neuroblastoma specimen. Additionally, we observed no significant difference between prediction probabilities for fresh frozen and FFPE samples. While we would normally expect higher-quality results from fresh frozen samples than FFPE, there is a compensatory effect since our prediction model is trained on predominantly FFPE samples (87%). These results suggest that we are able to model tumor-specific features contributing to accurate diagnosis that are preserved across both fresh-frozen and FFPE samples.

### FOXO1 fusion and MYCN amplification

The ability to classify tumor genomic subtypes simultaneously at the time of primary diagnosis has the potential to lead to avoidance of stepwise molecular testing, where it is available. Determining the *FOXO1* fusion status of rhabdomyosarcoma is an essential distinction to make given differences in disease prognosis and treatment regimens for fusion positive and fusion negative subtypes^23^. Fusion positive samples reflect chromosomal translocations t (1;13) or t (2;13), which correlate with *PAX7::FOXO1* and *PAX3::FOXO1* fusions respectively^23^. *FOXO1* fusions are correlated with more aggressive disease and poorer outcomes. Conversely, fusion negative specimens lack these fusions and are associated with more favorable clinical outcomes. Chemotherapeutic agent combination choices, prognostication, and treatment approaches differ by fusion status. *MYCN* oncogene amplification is the most important gene marker of neuroblastoma severity as it leads to unrestricted tumor growth and proliferation, indicating a poorer prognosis that requires a different treatment regimen compared to neuroblastoma without *MYCN* amplification^24^.

Previously reported differentially expressed genes are not robustly recapitulated in our model in part due to our filtering of genes that are not broadly expressed across our dataset. Low-coverage transcriptome sequencing results in a relatively sparse sampling of the transcriptome and, together with our previous work^17^, we show that our prediction models perform better and avoid overfitting when the majority of sparsely sequenced genes are excluded. We considered the expression of MYCN itself, which is expected to correlate with genomic MYCN amplification. MYCN expression is strongly correlated with FISH-based MYCN amplification status (Supplemental Fig. S8, Supplemental Table S5), but is excluded from our model because its observed expression is zero in 14 of 31 neuroblastoma samples with known MYCN amplification status (two neuroblastoma samples are not characterized). These 14 are all negative for MYCN amplification. This clear example of exclusion of a very strong marker gene based on the architecture of our model leaves the possibility of improving the model in the future if features like these can be included without contributing to over-fitting. In fact, a trivial heuristic model that stratifies our neuroblastoma samples by MYCN expression (normalized by expression of a housekeeping gene NAGK^25^, where ≥ 5 is considered MYCN amplification) produces slightly better aggregate results—90% accuracy—than our machine learning model.

### Cost effectiveness

Very low capital cost coupled with the ability to run multiplexed barcoded samples at multiple cost scales suggests that whole transcriptome sequencing of FFPE specimens for solid tumor diagnosis has the potential to reduce health costs and shorten time to complete diagnosis. Capital costs, including the MinION sequencer, operating computer, and basic equipment such as a PCR machine, total less than $5000 USD. Multiplexed, up to 12 samples can be run on one consumable MinION flow cell ($500–$1000) while ensuring adequate depth and throughput for each different specimen, bringing the cost of classifying each specimen to just under $100 including reagents. We utilized the higher capacity P2 sequencer ($10,000) for retrospective sequencing of up to 96 samples at once, but no differences other than throughput were observed across platforms. We previously established that suitable data is produced by a single flongle flow cell (~ $100)^17^, allowing for economies of scale and turnaround times to be matched to clinical needs. While the per-nucleotide sequencing costs of traditional next-generation sequencing-by-synthesis platforms (notably, Illumina) continue to drop and are typically lower than ONT sequencing, the capital costs for machines that achieve this economy of scale is orders of magnitude higher, and to achieve a similar cost point per sample, would require multiplexing many hundreds of samples simultaneously. The ability to run small batches with a short turnaround time is a critical consideration for potential molecular diagnostics applications. In-context implementation studies will be necessary to firmly establish the practical cost of this approach relative to standard of care molecular diagnostics, but the establishment of a nanopore sequencing-based solid tumor diagnosis assay has the potential to obviate the need for other cytologic and chromosomal tests in areas where they are unavailable, at a fraction of the cost.

### Quality control, validation, and implementation

Developing an implementation strategy at LMIC sites will allow for validation and setting of QC parameters for standardization of procedures while testing the robustness of this approach in diverse laboratory conditions. This will involve setting parameters such as prediction probability cutoffs that maximize accuracy, the minimum read N50 (50th percentile of cDNA read lengths) required, the read alignment rate, proportion of aligned reads, and Shannon entropy cutoff that are maximally discriminative for classification accuracy. Refining these QC criteria along with expansion of the training dataset promises to increase the accuracy and calibrated prediction probabilities of this approach in subsequent studies. Subsequent validation of the proposed approach and machine learning model will require additional cross-validation and independent validation cohort to assess possible overfitting/biases in this model, its extensibility to independent datasets, and potential variation in preparation and sequencing methodology. Only leave-one-out cross-validation was feasible in this study due to the limited sample size, especially in under-represented tumor types (ex. T-LL, DSRCT), however expansion of the training dataset will permit additional validation under more robust cross-validation splits.

### Future directions

This approach requires extensive knowledge in bioinformatics and genomics to operationalize in a routine clinical setting. This can be overcome in the future by integrating informatic processing and classification into user-friendly local or cloud computing infrastructure. Technical training is required for procedures including RNA extraction, RT-PCR, library preparation, and sequencing is setting with limited molecular biology experience. Further implementation and validation studies in resource-limited settings will help clarify technical and informatic barriers to adoption. Ongoing and future work in LMIC with additionally serve as orthogonal data to validate the performance of our proposed machine learning-based classifier. Sequencing additional normal/non-tumor tissues, especially infection-related growths commonly observed in low-resource settings, will improve our model’s ability to distinguish malignant from non-malignant tissue in clinically-relevant contexts. Continuous integration of additional sequenced samples into our machine learning model will continue to improve classification confidence and accuracy, especially across rarer tumor types.

Our results show that whole transcriptome sequencing-based classification of pediatric extracranial solid tumors and lymphomas may be applicable and practical in settings where the full spectrum of tests required for pediatric solid tumor diagnosis is inaccessible. Nanopore sequencing platforms represent a cost-effective and accessible technology to enable molecular cancer diagnostics in low-resource settings. We further demonstrated that RNA can be effectively extracted from FFPE specimens—the primary diagnostic sample available in many LMICs—and efficiently sequenced on nanopore platforms. The resulting expression profiles can discriminate common pediatric solid tumor and lymphomas, permitting timely diagnosis and assignment of appropriate treatment regimen that may correspondingly improve cancer outcomes and help bridge the cancer disparity gap between LMICs and HICs.

### Supplementary Information

Supplementary Figures.

Supplementary Tables.

## Data Availability

The datasets generated and analyzed as a part of the current study are available via controlled access at 10.5281/zenodo.11245094. Sample metadata are available in Supplemental Table S1. Classification results and prediction probabilities are available in Supplemental Tables S2 and S3.

## References

[1] Ren, H.-M. *et al.* Global, regional, and national burden of cancer in children younger than 5 years, 1990–2019: Analysis of the global burden of disease study 2019. *Front. Public Health* **10**, 910641. 10.3389/fpubh.2022.910641 (2022). 35801252 10.3389/fpubh.2022.910641 PMC9255714

[2] Wu, Y. *et al.* Global, regional, and national childhood cancer burden, 1990–2019: An analysis based on the Global Burden of Disease Study 2019. *J. Adv. Res.* **40**, 233–247. 10.1016/j.jare.2022.06.001 (2022). 35700919 10.1016/j.jare.2022.06.001 PMC9481947

[3] Fung, A., Horton, S., Zabih, V., Denburg, A. & Gupta, S. Cost and cost-effectiveness of childhood cancer treatment in low-income and middle-income countries: A systematic review. *BMJ Glob. Health* **4**(5), 1. 10.1136/bmjgh-2019-001825 (2019). 10.1136/bmjgh-2019-001825 PMC6830048 31749998

[4] Renner, L. *et al.* Evidence from Ghana indicates that childhood cancer treatment in sub-Saharan Africa is very cost effective: A report from the childhood cancer 2030 network. *J. Glob. Oncol.* **4**, 1–9. 10.1200/JGO.17.00243 (2018). 30241273 10.1200/JGO.17.00243 PMC6223505

[5] Johnston, W. T. *et al.* Childhood cancer: Estimating regional and global incidence. *Cancer Epidemiol.* **71**(Pt B), 101662. 10.1016/j.canep.2019.101662 (2021). 31924557 10.1016/j.canep.2019.101662

[6] Verma, N. & Bhattacharya, S. Time to diagnosis and treatment of childhood cancer. *Indian J. Pediatr.* **87**(8), 641–643. 10.1007/s12098-020-03217-y (2020). 32056193 10.1007/s12098-020-03217-y

[7] Bhakta, N. *et al.* Childhood cancer burden: A review of global estimates. *Lancet Oncol.* **20**(1), e42–e53. 10.1016/S1470-2045(18)30761-7 (2019). 30614477 10.1016/S1470-2045(18)30761-7

[8] American Cancer Society. (n.d.). *Ewing Tumor—Tests|American Cancer Society*. Retrieved April 5, 2023. https://www.cancer.org/cancer/ewing-tumor/detection-diagnosis-staging/how-diagnosed.html.

[9] Gupta, S., Howard, S. C., Hunger, S. P., Antillon, F. G., Metzger, M. L., Israels, T., Harif, M., & Rodriguez-Galindo, C. Treating childhood cancer in low- and middle-income countries. In Gelband, H. (Eds.) Cancer: Disease control priorities, Third Edition (Volume 3). The International Bank for Reconstruction and Development/The World Bank (2015). 26913338

[10] Marino, P. *et al.* Cost of cancer diagnosis using next-generation sequencing targeted gene panels in routine practice: A nationwide French study. *Eur. J. Hum. Genet.* **26**(3), 314–323. 10.1038/s41431-017-0081-3 (2018). 29367707 10.1038/s41431-017-0081-3 PMC5838982

[11] Tuna, M. & Amos, C. I. Genomic sequencing in cancer. *Cancer Lett.* **340**(2), 161–170. 10.1016/j.canlet.2012.11.004 (2013). 23178448 10.1016/j.canlet.2012.11.004 PMC3622788

[12] Wang, L. & Wheeler, D. A. Genomic sequencing for cancer diagnosis and therapy. *Annu. Rev. Med.* **65**, 33–48. 10.1146/annurev-med-120811-171056 (2014). 24274147 10.1146/annurev-med-120811-171056

[13] Cuppen, E., Elemento, O., Rosenquist, R. & Nikic, S. Implementation of whole-genome and transcriptome sequencing into clinical cancer care. *JCO Precis. Oncol.* **6**, e2200245. 10.1200/PO.22.00245 (2022). 36480778 10.1200/PO.22.00245 PMC10166391

[14] Mimosa, M. L. *et al.* A novel approach to detect IDH point mutations in gliomas using nanopore sequencing: Test validation for the clinical laboratory. *J. Mol. Diagn. JMD* **25**(3), 133–142. 10.1016/j.jmoldx.2022.12.001 (2023). 36565986 10.1016/j.jmoldx.2022.12.001

[15] Tan, O., Shrestha, R., Cunich, M. & Schofield, D. J. Application of next-generation sequencing to improve cancer management: A review of the clinical effectiveness and cost-effectiveness. *Clin. Genet.* **93**(3), 533–544. 10.1111/cge.13199 (2018). 29265354 10.1111/cge.13199

[16] Sequencing short fragments with nanopore technology (n.d.). Oxford Nanopore Technologies. Retrieved June 26, 2023, from https://nanoporetech.com/applications/techniques/short-fragment-mode.

[17] Wang, J. *et al.* Acute leukemia classification using transcriptional profiles from low-cost nanopore mRNA sequencing. *JCO Precis. Oncol.* **6**, e2100326. 10.1200/PO.21.00326 (2022). 35442720 10.1200/PO.21.00326 PMC9200386

[18] Cunningham, F., Allen, J. E., Allen, J., Alvarez-Jarreta, J., Amode, M. R., Armean, I. M., Austine-Orimoloye, O., Azov, A. G., Barnes, I., Bennett, R., Berry, A., Bhai, J., Bignell, A., Billis, K., Boddu, S., Brooks, L., Charkhchi, M., Cummins, C., Da Rin Fioretto, L., & Flicek, P. Ensemble 2022. *Nucleic Acids Res.* **50**(D1), D988–D995. 10.1093/nar/gkab1049 (2022). 10.1093/nar/gkab1049 PMC8728283 34791404

[19] Li, H. Minimap2: Pairwise alignment for nucleotide sequences. *Bioinformatics* **34**(18), 3094–3100. 10.1093/bioinformatics/bty191 (2018). 29750242 10.1093/bioinformatics/bty191 PMC6137996

[20] Shannon, C. E. A mathematical theory of communication. *Bell Syst. Tech. J.* **27**, 379–423 (1948).

[21] Williamson, D. *et al.* Fusion gene-negative alveolar rhabdomyosarcoma is clinically and molecularly indistinguishable from embryonal rhabdomyosarcoma. *JCO* **28**, 2151–2158 (2010). 10.1200/JCO.2009.26.3814 20351326

[22] Schramm, A. *et al.* Next-generation RNA sequencing reveals differential expression of MYCN target genes and suggests the mTOR pathway as a promising therapy target in MYCN-amplified neuroblastoma. *Int. J. Cancer* **132**, E106–E115 (2013). 22907398 10.1002/ijc.27787

[23] Heske, C. M. *et al.* Survival outcomes of patients with localized FOXO1 fusion positive rhabdomyosarcoma treated on recent clinical trials: A report from the Soft Tissue Sarcoma Committee of the Children’s Oncology Group. *Cancer* **127**(6), 946–956. 10.1002/cncr.33334 (2021). 33216382 10.1002/cncr.33334 PMC8601034

[24] Kaczówka, P. *et al.* The role of N-Myc gene amplification in neuroblastoma childhood tumour—single-centre experience. *Contemp. Oncol. (Poznan, Poland)* **22**(4), 223–228. 10.5114/wo.2018.81402 (2018). 10.5114/wo.2018.81402 PMC6377415 30783385

[25] Gotoh, T. *et al.* Prediction of MYCN amplification in neuroblastoma using serum DNA and real-time quantitative polymerase chain reaction. *J. Clin. Oncol.* **23**, 5205–5210 (2005). 16051962 10.1200/JCO.2005.02.014

