# Supplementary material for: Transcriptome profiling of pediatric extracranial solid tumors and lymphomas enables rapid low-cost diagnostic classification
Source: Sci Rep. 2024 Aug 21;14:19456. doi: 10.1038/s41598-024-70541-0 (PMC11339337; doi:10.1038/s41598-024-70541-0)
Supplement: Supplementary file 1 — Supplementary Figures. [file 41598_2024_70541_MOESM1_ESM.docx]

**Supplemental materials**

Opoku *et al*. Transcriptome profiling of pediatric extracranial solid tumors and lymphomas enables rapid low-cost diagnostic classification.

**Supplemental Figure 1.** Correlation between Shannon entropy and prediction probability. Each point is a classified sample where green are correctly called tumor types and red are incorrect. We see a statistically significant relationship (*p* = 0.0015) between prediction probability and Shannon entropy, albeit of small effect (*r^2^* = 0.0393).


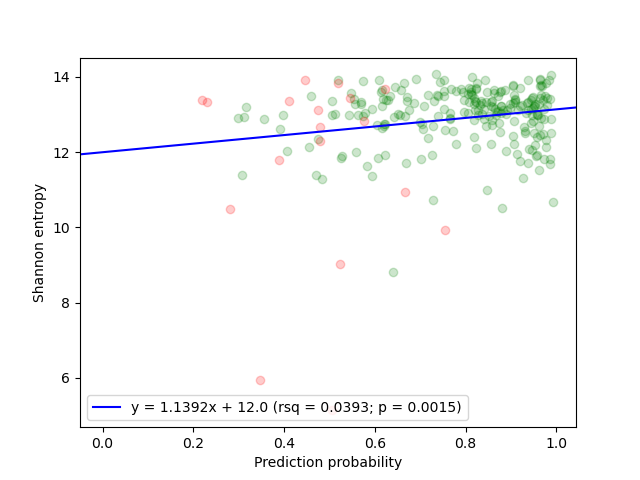


**Supplemental Figure 2.** Correlation between therapy status (pre- or post-chemotherapy, where 0 is pre-therapy and 1 is post-therapy) and prediction probability. Each point is a classified sample where green are correctly called tumor types and red are incorrect. We observe no significant correlation between prediction probability and therapy status (*p* = 0.6804).


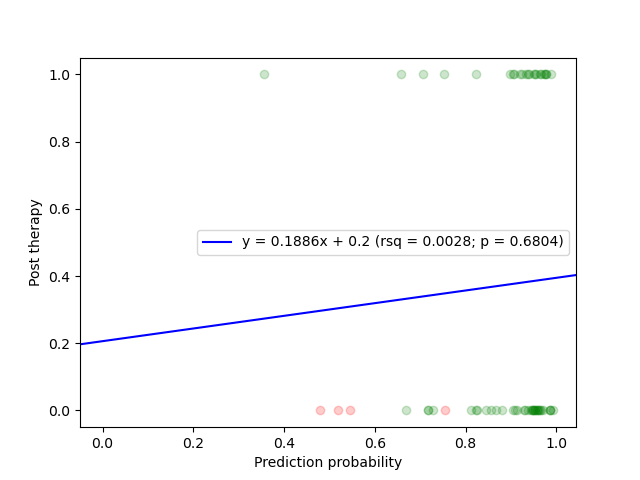


**Supplemental Figure 3.** Correlation between tumor purity (% tumor vs. normal cells) and prediction probability. Each point is a classified sample where green are correctly called tumor types and red are incorrect. We observe no significant correlation between prediction probability and therapy status (*p* = 0.1968).


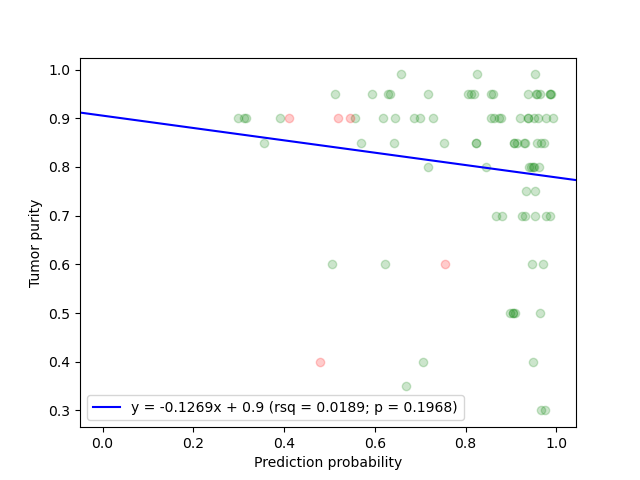


**Supplemental Figure 4.** Previously published differentially expressed genes by *FOXO1* fusion status [Williamson, 2009]. Mann-Whitney U test and found none are significantly differentially expressed in our data after correcting for multiple testing.


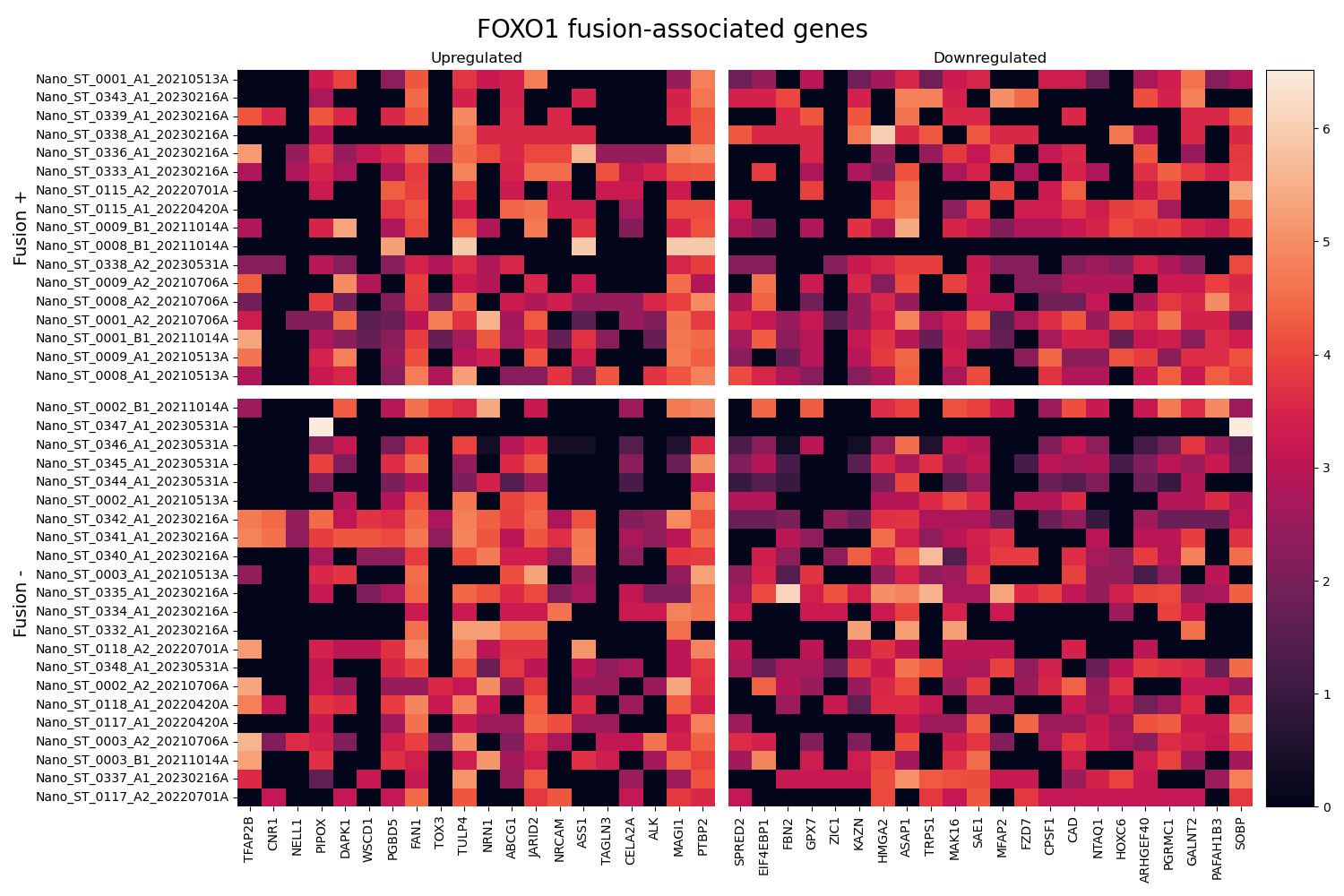


**Supplemental Figure 5.** Highest weighted gene features discriminating *FOXO1* fusion status in our nanopore RNAseq expression profiles.


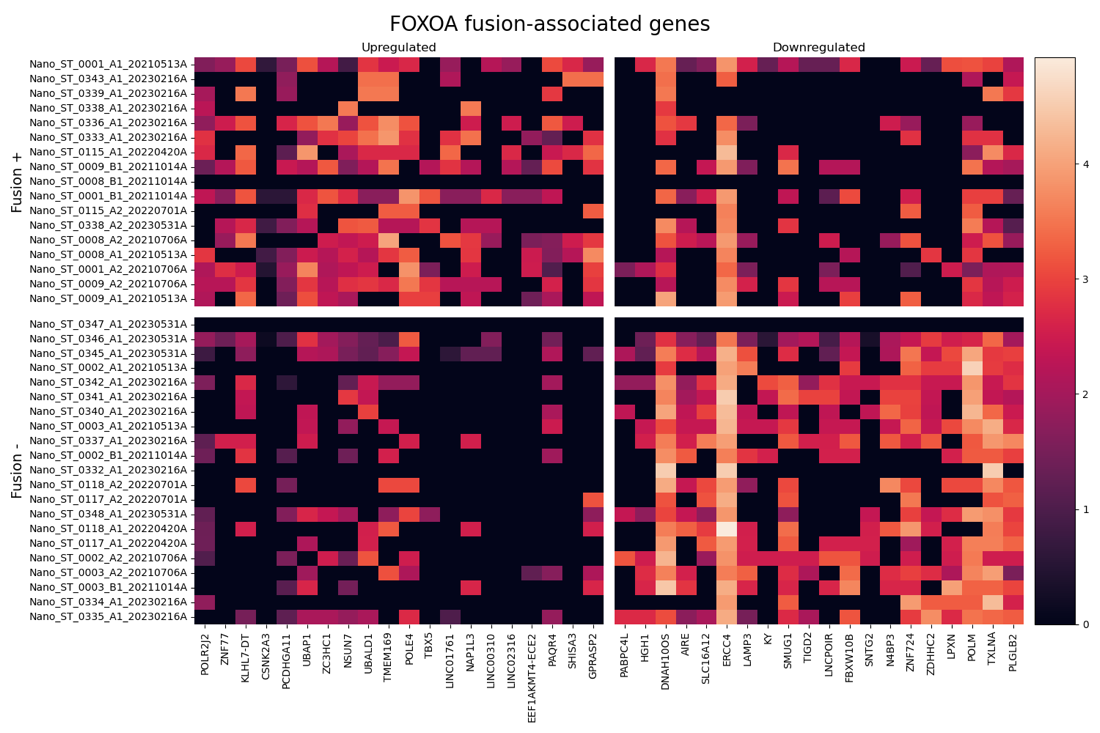


**Supplemental Figure 6.** Previously published differentially expressed genes by *MYCN* amplification status [Schramm, 2012]. None show a significant correlation with *MYCN* amplification status in our cohort using nanopore RNAseq data.


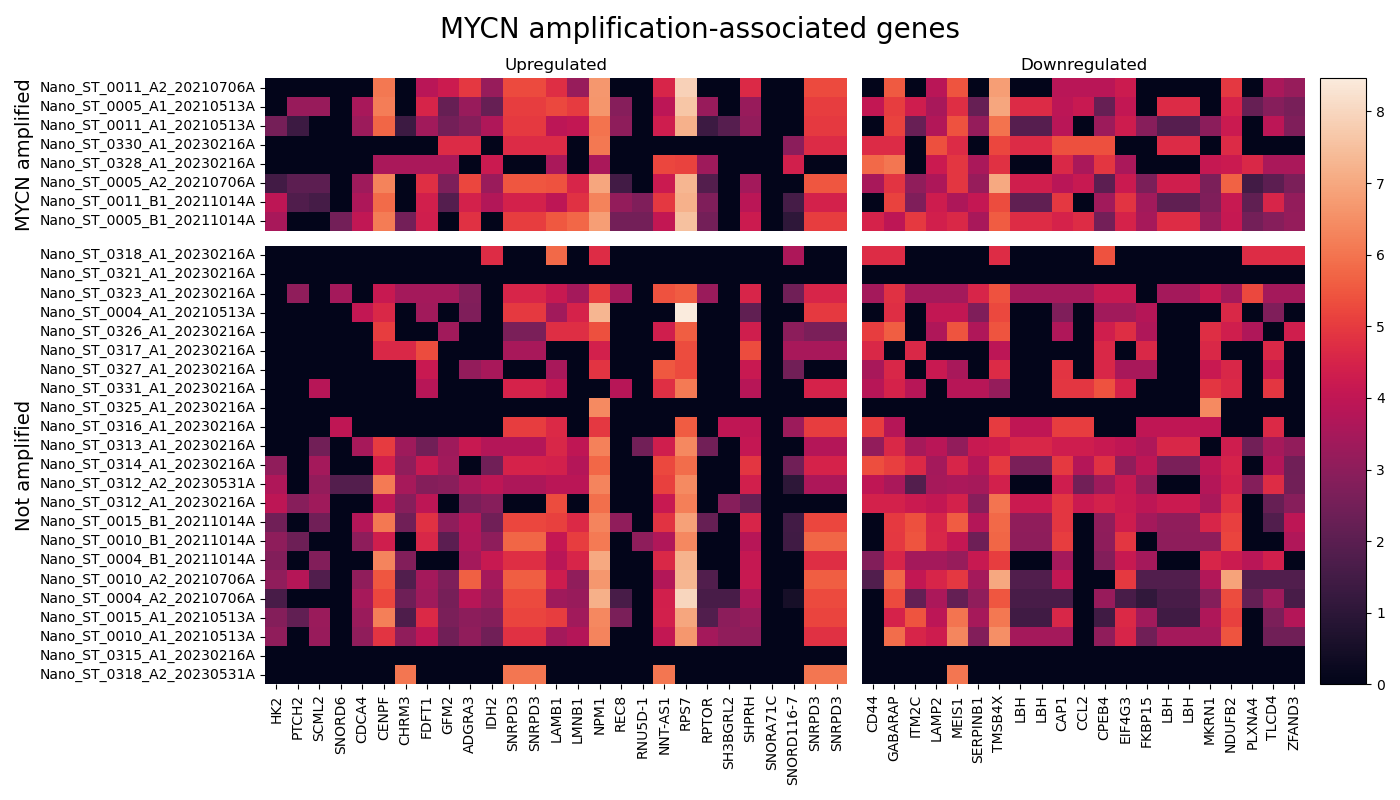


**Supplemental Figure 7.** Highest weighted gene features by *MYCN* status in our nanopore RNAseq expression profiles.


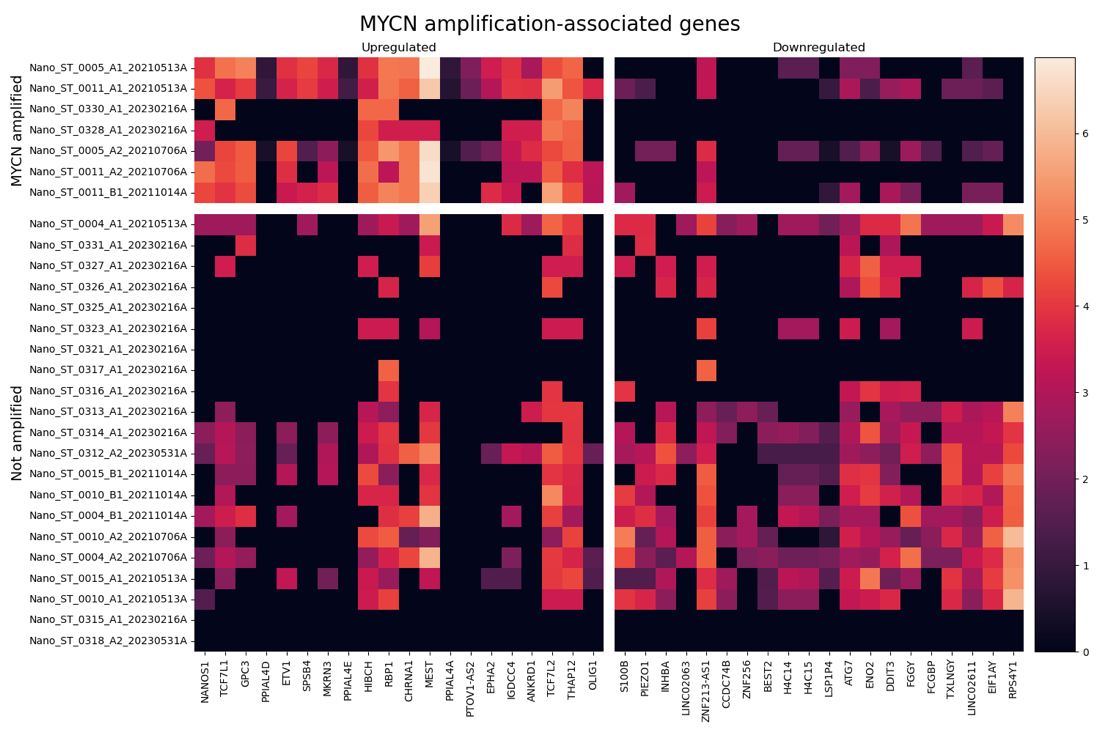


**Supplemental Figure 8.** *MYCN:NAGK* expression by *MYCN* amplification status among neuroblastoma samples. *MYCN* expression is shown relative to *NAGK* control for neuroblastoma specimens colored by *MYCN* amplification status (red: amplified, blue: unamplified). Relative *MYCN* expression alone permits ~90% accurate stratification of specimens by *MYCN* amplification status with a cutoff of 5.


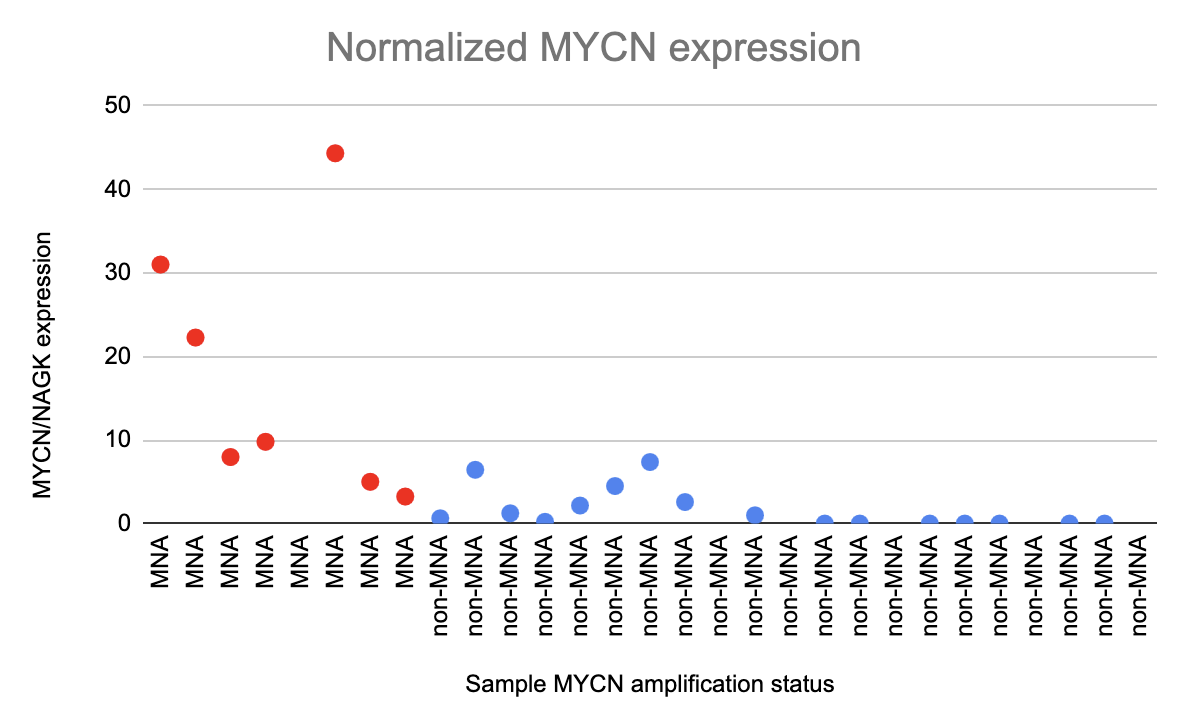


**Supplemental Figure 9.** Confusion matrix showing nanopore transcriptome-based classification results compared to standard of care clinical diagnosis for pediatric solid tumors (A) and lymphomas (B).

A)
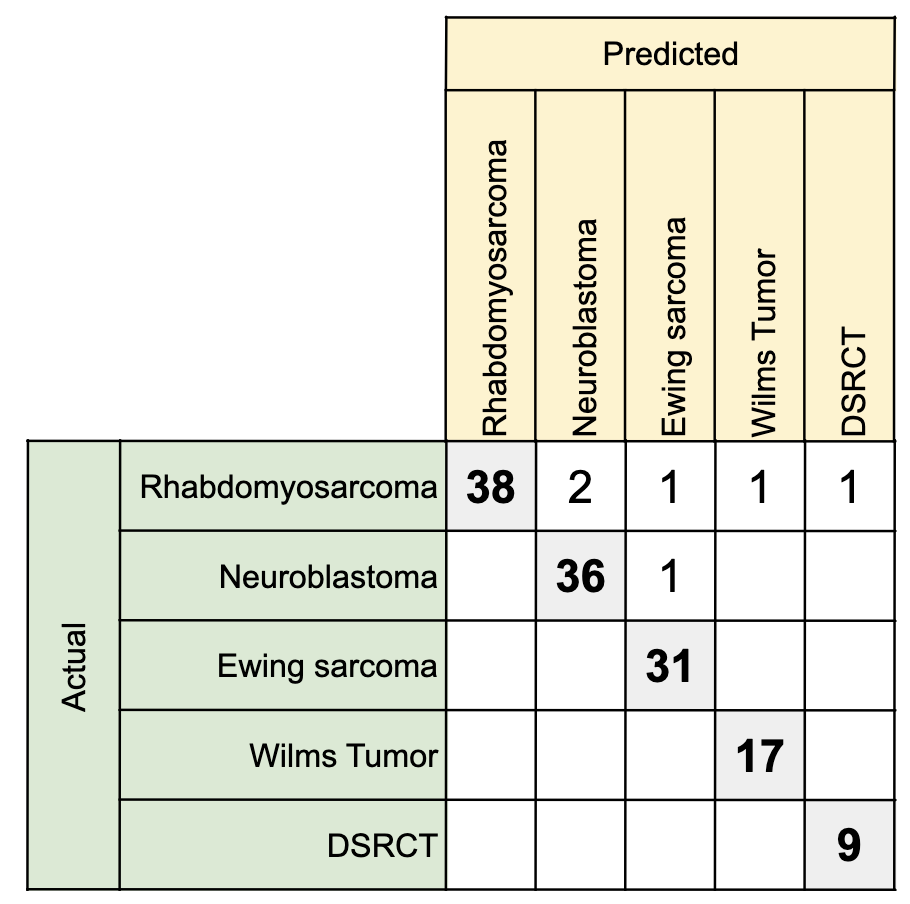


B)
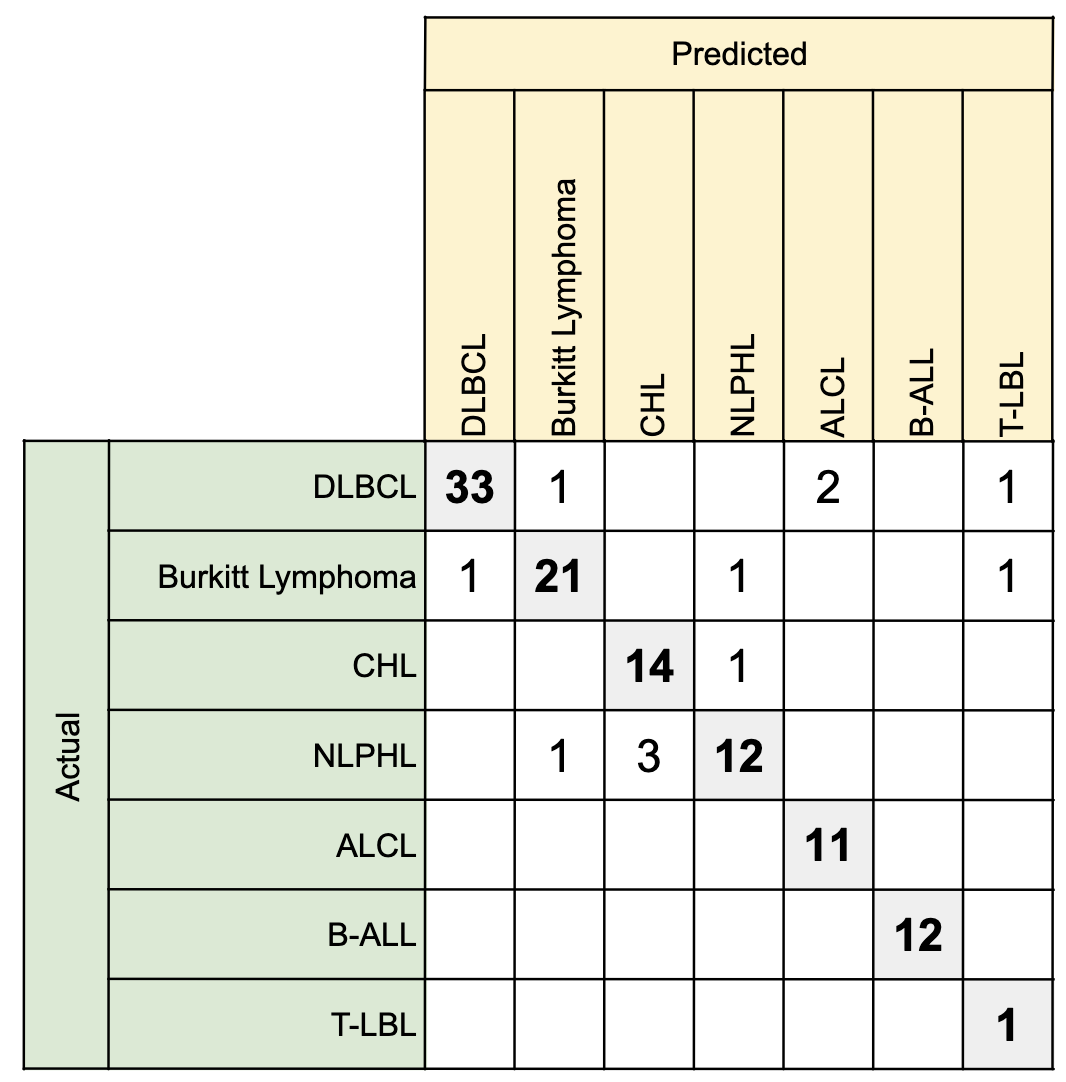


**Supplemental Figure 10.** Principal component analysis of gene expression among pediatric solid tumor (A) and lymphoma (B) specimens, colored by batch (processing and sequencing date) shows minimal batch effects. Lymphoma batch 20221110A shows slight separation along the minor principal component (5.21% of variation) and is correlated with Hodgkin lymphoma (CHL and NLPHL) cases.

A)
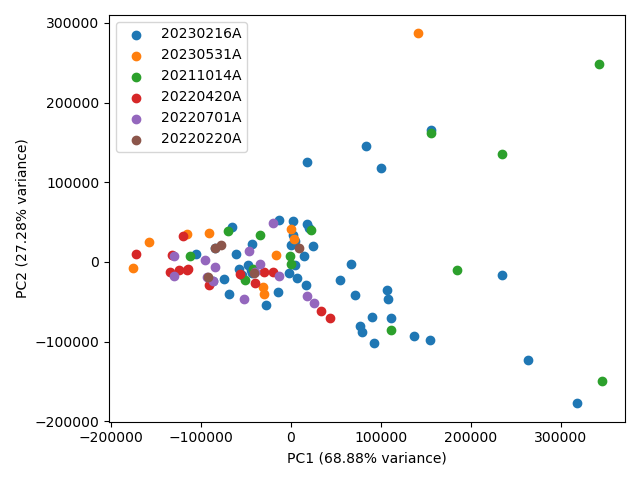


B)
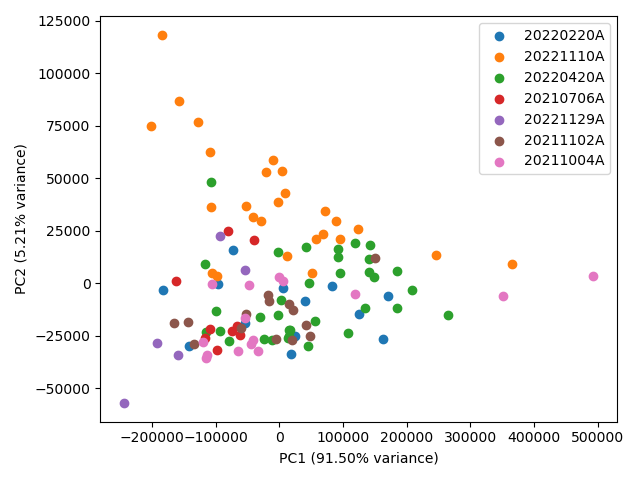


**Supplemental Table 1.** Sample list and clinical metadata

**Supplemental Table 2.** Solid tumor classification results with prediction probabilities

**Supplemental Table 3**. Lymphoma classification results with prediction probabilities

**Supplemental Table 4.** Sequencing data statistics, assigned genes, and entropy

**Supplemental Table 5.** *MYCN* amplification status classification results and comparison to naive *MYNC* expression model

**Supplemental Table 6.** Gene expression profiles in transcripts per million (TPM)
